# Trends in survival for cancer patients aged 65 years or over from 1995 to 2014 in the United States: A population‐based study

**DOI:** 10.1002/cam4.5398

**Published:** 2022-11-10

**Authors:** Lan An, Wen Ju, Rongshou Zheng, Hongmei Zeng, Siwei Zhang, Ru Chen, Kexin Sun, Li Li, Shaoming Wang, Wenqiang Wei

**Affiliations:** ^1^ National Central Cancer Registry, National Cancer Center/National Clinical Research Center for Cancer/Cancer Hospital Chinese Academy of Medical Sciences and Peking Union Medical College Beijing China

**Keywords:** epidemiology and prevention, prognosis, SEER, survival

## Abstract

**Background:**

Adults aged 65 years and above account for over half of all cancer diagnoses in the United States, but little is known about trend of elderly cancer survival in the United States. We aimed to assess the survival trend for elderly cancer in the United States from 1995 to 2014.

**Methods:**

We used data from Surveillance, Epidemiology, and End Results 12 registries and included 1,112,441 eligible patients aged 65 years or older who were diagnosed between 1995 and 2014 with cancer and followed up until December 2019. Overall and stage‐specific 5‐year relative survival, ratio of observed survival to expected survival, with 95% confidence intervals (CIs) of elderly cancer patients stratified by age were estimated during four periods (1995–1999, 2000–2004, 2005–2009, and 2010–2014). Cox proportional hazards models were used to estimate hazard ratios for cancer‐specific death among patients diagnosed during 2000–2004, 2005–2009, 2010–2014, compared diagnoses in 1995–1999. We also calculated stage distribution and treatment rate during four periods.

**Results:**

In the United States, 5‐year relative survival for elderly cancer patients improved from 57.3% (95% CIs 57.0–57.5) in 1995–1999 to 60.7% (60.5–60.9) in 2010–2014. After controlling for sociodemographic and tumor characteristics, about a 19% reduction in cancer‐specific deaths among diagnoses in 2010–2014 compared with 1995–1999. Cancer survival improved for elderly patients in all age groups, with exception of stable survival for patients aged 85 and above. Comparing 1995–1999 with 2010–2014, relative survival improved from 84.7% (84.3–85.1) to 86.7% (86.3–87.0) for localized stage and from 12.4% (12.1–12.7) to 18.7% (18.4–19.0) for distant stage for all cancers combined. The trends in stage distribution and treatment rate for all cancers combined were relatively stable.

**Conclusions:**

In the United States, survival for elderly cancer patients has improved slightly from 1995 to 2014, possibly mainly due to advances in treatment. Further studies are warranted to explore interventions to improve elderly cancer survival.

## Graphical abstract

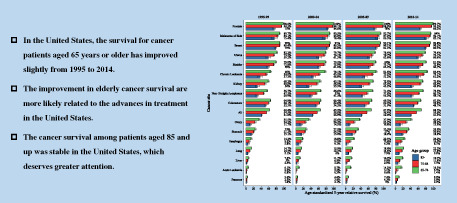

## 1 INTRODUCTION

Cancer is primarily a disease of the elderly, with patients aged 65 years or older accounting for over half of new cancer diagnoses and more than two thirds of cancer‐specific deaths in the United States.^1^ Because of increased life expectancy and an aging population, the number of elderly cancer cases is expected to rise further.^2^ Therefore, the burden of elderly cancer patients has become one of the country's most pressing public health issues. Given high level of comorbidity, reduced functional status, late‐stage at diagnosis because of less screening, and less aggressive treatment, the elderly patients have lower cancer survival relative to younger patients.^3^, ^4^, ^5^, ^6^ Previous study found a slower improvement in cancer survival for older patients than for younger patients in the United States from 1990 to 2010, resulting in a growing survival difference between the young and the old, in part because the elderly patients do not gain as much from developments in cancer therapies as younger patients.^5^ It has been reported that the overall cancer survival has been improved from 57.7% in 2000 to 59.8% in 2013 among patients aged 65 and above.^7^ However, the trend of elderly cancer survival in the United States from 1995 to 2014, especially among various age groups, is not well understood. Additionally, it is essential to identify the underlying causes of trends in elderly cancer survival for targeted cancer control measures aimed at improving cancer outcome.

Increased life expectancy may account for part of the improvement in survival in older age brackets. Relative survival, the ratio of cancer patients' observed survival to the expected survival of a comparable group from the general population, accounts for changes in background mortality over time (as determined by general population life tables) and is therefore focused on survival from cancer.^8^ In addition, considering multiple prognostic factors, assessment of cancer‐specific death between calendar periods could quantify the degree of trend in survival for elderly cancer patients. Therefore, this study aims to use two indicators to assess the survival estimates and trends of elderly cancer patients overall and by age group between 1995 and 2014 in the United States, using data from the most recent population‐based cancer registries. This study examined the trend of stage distribution, treatment rate and stage‐specific survival to explore the potential causes of such trends and differences. These findings could provide scientific evidence for resource allocation of elderly cancer patients, as well as intervention to narrow these disparities.

## 2 MATERIALS AND METHODS

### 2.1 Data sources

The data was obtained from the US National Cancer Institute's Surveillance, Epidemiology, and End Results (SEER) 12 registries research database.^9^ This database contains data from the 12 SEER areas (the metropolitan areas of Atlanta; Connecticut; San Jose‐Monterey; Los Angeles; Alaska Natives; Rural Georgia; Hawaii; Iowa; New Mexico; San Francisco‐Oakland, California; Seattle‐Puget Sound, Washington; and Utah), and represents about 12.2% of the total US population.

We identified elderly patients (65 years or over) with first primary diagnoses of cancer using the third edition of the International Classification of Disease for Oncology for all cancers and 16 major cancer types, including acute leukemia, bladder, chronic leukemia, colon/rectum, corpus and uterus (“uterus”), esophagus, female breast, kidney, liver/intrahepatic bile duct (“liver”), lung, melanoma of skin, non‐Hodgkin lymphoma (“NHL”), ovary, pancreas, prostate, and stomach. The 16 cancer types accounts for around 85% of all elderly cancer cases. We chose patients diagnosed from 1995 to 2014 and followed up until December 31, 2019, with a minimum follow‐up period of 5 years. Patients aged less than 65 years, patients diagnosed by death certificate or autopsy, patients with unknown status, and patients with in situ tumors were excluded. Covariates included demographic variables (sex, race, age at diagnosis, rural–urban area, and marital status) and diagnostic information (year of diagnosis, stage at diagnosis, site, tumor grade, and common histology). Age at diagnosis was divided into three groups (65–74 years, 75–84 years, 85 years and above). Race categories included white, black, and other (American Indian/Alaskan Native, Asian/Pacific Islander, and unknown). Stage referred as SEER historic stage, grouping patients into localized, regional, distant, and unknown stage. Urban area was defined as counties in metropolitan areas, while rural area was defined as nonmetropolitan areas.

To compare survival in the United States with other countries, we extracted cancer survival from the IARC website based on the International Cancer Benchmarking Partnership (ICBP) SURVMARK project, which contains survival estimates based on high‐quality data from 21 population‐based cancer registries in seven high‐income countries (Australia, Canada, Denmark, Ireland, New Zealand, Norway, and the United Kingdom).^3^, ^10^

### 2.2 Statistical analysis

Relative survival was defined as the ratio of cancer patients' observed survival to the expected survival of a comparable group from the general population. We calculated five‐year relative survival with corresponding 95% confidence intervals (CIs) of elderly cancer patients by using SEER*Stat software program (version 8.4.0).^11^ The Ederer II approach was used to estimate expected survival using life tables stratified by socioeconomic status, geography, race, age, gender, and calendar year. We summarized overall and stage‐specific 5‐year relative survival by age group for patients diagnosed between 1995 and 1999, 2000 and 2004, 2005 and 2009, and 2010 and 2014.

Cancer‐specific death was defined as death from specific cancer in the SEER registries. Cox proportional hazards models were used to calculate hazard ratios (HRs) and corresponding 95% CIs for cancer‐specific death associated with age for patients diagnosed during the periods 2000 to 2004, 2005 to 2009, and 2010 to 2014, and were compared with those diagnosed during the baseline period (1995 to 1999). Survival time was defined as the time from the date of diagnosis until cancer‐specific death, date of last contact or end of study period (December 31, 2019) in months. Cases were censored if patients still alive on December 31, 2019, lost to follow‐up, or died of other causes. To assess the average cancer‐specific mortality from 1995 to 2014, we estimated HRs and 95% CIs for each 5‐year increment by year of diagnosis. Potential confounders, including sex, race, age at diagnosis, and rural–urban area, tumor grade, marital status, common histology, and stage at diagnosis, were included in Cox model. Visual examination of Kaplan–Meier curves for each variable in the study revealed no violation of the proportional hazards assumption. Considering the large sample size and numerous tests performed in our study, we adopted two‐sided *p* < 0.001 as statistically significant.

In addition, we examined the distribution of stage at diagnosis from SEER database and treatment rate stratified by the four calendar periods. As a sensitivity analysis, we analyzed the stage distribution of cancer patients with unknown stage, treating the unknown stage group as a separate group. The treatment variable was defined as the acceptance of any type of treatment including surgery, chemotherapy or radiation, during the first course of the therapy. Because the treatment variable had been collected since 1998, the period of treatment was divided into 1998–1999, 2000–2004, 2005–2009, and 2010–2014. We excluded non‐Hodgkin lymphoma and leukemia from the calculation of the proportion of stage. Data analysis was performed using SAS version 9.4 (SAS Institute, Cary, NC, US). We performed figures using R software version 4.1.2 (R Core Team, Vienna, Austria).

## 3 RESULTS

We identified a total of 1,112,441 eligible patients aged 65 years and above who had been diagnosed with cancer in the 12 SEER registries from 1995 through 2014. The number of elderly cancer patients overall, by age group and site are shown in Table S1.

The 5‐year relative survival for elderly cancer patients varied considerably by cancer site, ranging from 7.1% (95% CI 6.6–7.9) for pancreatic cancer to 97.0% (96.6–97.4) for prostate cancer in 2010–14 (Table 1). The 5‐year relative survival for all cancers combined increased from 57.3% (57.0–57.5) in 1995–1999 to 60.7% (60.5–60.9) in 2010–14. Except for cancers of the bladder, colorectum, and uterus, 5‐year relative survival increased steadily over time for the majority of cancer types. After controlling for sociodemographic and tumor characteristics, there was about a 19% reduction in cancer‐specific deaths among patients diagnosed between 2010 and 2014 [HR and 95% CI: 0.81(0.80–0.82)], compared with patients diagnosed between 1995 and 1999 (Table 2). Each cancer type evaluated in this study experienced significant improvement in survival over the 20‐year period, as evidenced by decreasing HRs over time. Chronic leukemia had the largest improvement in survival, followed by esophageal cancer, melanoma of skin and liver cancer [HR for 5‐year increment of year of diagnosis ranging from 0.76(0.74–0.78) for chronic leukemia to 0.97(0.95–0.98) for ovary]. Furthermore, compared to those diagnosed between 1990 and 1994, the improvement in survival for most cancer types began from 1995 to 1999, whereas the improvement in survival for ovarian cancer occurred later and was visible among cancer diagnoses after 2010.

**TABLE 1:** Trends in 5‐year relative survival by cancer site in elderly patients

Site	5‐year relative survival			
	1995–99	2000–04	2005–09	2010–14
Acute Leukemia	3.7 (3.0, 4.5)	4.5 (3.8, 5.4)	7.2 (6.2, 8.3)	9.3 (8.2, 10.5)
Bladder	74.9 (73.8, 75.9)	75.2 (74.2, 76.2)	74.6 (73.6, 75.6)	74.9 (73.9, 75.8)
Breast	88.7 (88.1, 89.3)	89.6 (89.0, 90.2)	90.2 (89.6, 90.7)	90.8 (90.3, 91.3)
Chronic Leukemia	57.7 (55.7, 59.6)	67.3 (65.4, 69.1)	73.1 (71.4, 74.7)	77.7 (76.0, 79.2)
Colorectum	60.8 (60.2, 61.5)	62.5 (61.9, 63.2)	63.3 (62.6, 64.0)	61.1 (60.4, 61.8)
Uterus	77.4 (76.1, 78.6)	76.7 (75.3, 78.0)	74.5 (73.2, 75.8)	76.0 (74.9, 77.1)
Esophagus	11.6 (10.4, 13.0)	16.1 (14.7, 17.6)	16.6 (15.2, 18.1)	19.7 (18.2, 21.2)
Kidney	54.1 (52.5, 55.6)	59.1 (57.7, 60.6)	66.4 (65.1, 67.7)	68.0 (66.8, 69.2)
Liver	6.0 (5.2, 6.8)	9.1 (8.2, 10.1)	12.4 (11.4, 13.3)	15.6 (14.7, 16.6)
Lung	12.4 (12.1, 12.8)	13.5 (13.1, 13.8)	15.8 (15.4, 16.2)	18.9 (18.5, 19.3)
Non‐Hodgkin lymphoma	49.8 (48.7, 51.0)	58.2 (57.1, 59.3)	62.0 (60.9, 63.0)	65.1 (64.1, 66.1)
Ovary	28.3 (26.8, 29.8)	28.8 (27.3, 30.3)	27.7 (26.3, 29.2)	33.5 (32.0, 35.1)
Pancreas	2.9 (2.5, 3.3)	3.7 (3.3, 4.2)	5.3 (4.8, 5.8)	7.1 (6.6, 7.6)
Prostate	96.1 (95.6, 96.5)	98.7 (98.2, 99.0)	98.5 (98.0,98.8)	97.0 (96.6, 97.4)
Melanoma of Skin	86.5 (85.0, 87.9)	87.6 (86.2, 88.9)	89.9 (88.7, 91.1)	92.2 (91.2, 93.1)
Stomach	21.9 (20.8, 23.0)	24.5 (23.3, 25.7)	28.2 (26.9, 29.5)	30.9 (29.6, 32.2)
All	57.3 (57.0, 57.5)	59.4 (59.2, 59.6)	60.4 (60.2, 60.6)	60.7 (60.5, 60.9)

**TABLE 2:** Multivariate‐adjusted hazard ratios (HRs) and 95% confidence intervals (CIs) for cancer specific death associated with year of diagnosis, 12 SEER registries, 1995–2014

Site	1995–99	2000–04	2005–09	2010–14	Per 5‐year increment
Acute Leukemia	1.00	0.95 (0.90–1.00)	0.84 (0.79–0.89)	0.74 (0.70–0.79)	0.90 (0.89–0.92)
Bladder	1.00	0.94 (0.90–0.99)	0.96 (0.91–1.01)	0.86 (0.82–0.90)	0.96 (0.94–0.97)
Breast	1.00	0.90 (0.86–0.93)	0.82 (0.79–0.86)	0.74 (0.71–0.77)	0.91 (0.90–0.92)
Chronic Leukemia	1.00	0.77 (0.72–0.82)	0.58 (0.54–0.62)	0.44 (0.40–0.47)	0.76 (0.74–0.78)
Colorectum	1.00	0.95 (0.92–0.97)	0.87 (0.85–0.89)	0.85 (0.83–0.87)	0.94 (0.94–0.95)
Uterus	1.00	0.96 (0.90–1.03)	0.94 (0.88–1.01)	0.82 (0.77–0.88)	0.94 (0.92–0.96)
Esophagus	1.00	0.83 (0.78–0.89)	0.75 (0.70–0.80)	0.65 (0.60–0.69)	0.87 (0.85–0.89)
Kidney	1.00	0.90 (0.85–0.95)	0.79 (0.74–0.83)	0.73 (0.69–0.77)	0.90 (0.88–0.91)
Liver	1.00	0.90 (0.85–0.95)	0.79 (0.75–0.84)	0.68 (0.65–0.72)	0.88 (0.87–0.89)
Lung	1.00	0.93 (0.91–0.94)	0.82 (0.81–0.84)	0.75 (0.74–0.77)	0.91 (0.90–0.91)
Non‐Hodgkin lymphoma	1.00	0.90 (0.85–0.95)	0.79 (0.74–0.83)	0.73 (0.69–0.77)	0.90 (0.88–0.91)
Ovary	1.00	1.00 (0.95–1.05)	1.00 (0.95–1.06)	0.90 (0.85–0.94)	0.97 (0.95–0.98)
Pancreas	1.00	0.92 (0.89–0.95)	0.83 (0.81–0.86)	0.77 (0.74–0.79)	0.91 (0.90–0.92)
Prostate	1.00	0.82 (0.80–0.85)	0.75 (0.72–0.77)	0.74 (0.71–0.76)	0.89 (0.88–0.91)
Melanoma of Skin	1.00	0.87 (0.80–0.94)	0.85 (0.78–0.92)	0.67 (0.61–0.72)	0.88 (0.86–0.91)
Stomach	1.00	0.93 (0.89–0.97)	0.82 (0.78–0.85)	0.74 (0.71–0.77)	0.90 (0.89–0.92)
All	1.00	0.95 (0.94–0.96)	0.88 (0.87–0.89)	0.81 (0.80–0.82)	0.93 (0.93–0.93)

**FIGURE 1:**
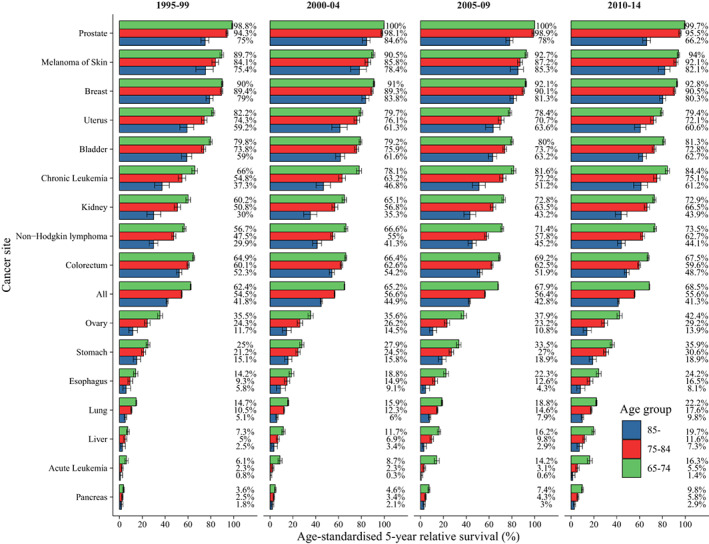
Trends in 5‐year relative survival in elderly patients, by age group and cancer site.

To dig deeper into the survival profiles, trends in survival by age group for all cancers combined as well as 16 individual cancers were analyzed. For all cancer sites combined, 5‐year relative survival declined as age group [68.5% (68.3–68.8) in 65–74 years, 55.6% (55.2–56.0) in 75–84 years, and 41.3% (40.6–42.1) in 85‐years] in 2010–14 (Figure 1). Considering a number of prognostic factors, cancer patients aged 65–74 years [HR for 5‐year increment of year of diagnosis: 0.89 (0.88–0.89)] had a greater increase in survival than patients aged 75–84 years [0.96 (0.95–0.96)] from 1995 to 2014. Overall cancer survival did not improve in patients aged 85 and older, but survival from leukemia, lung cancer, and liver cancer improved in patients aged 85 and older (Table S2).

**FIGURE 2:**
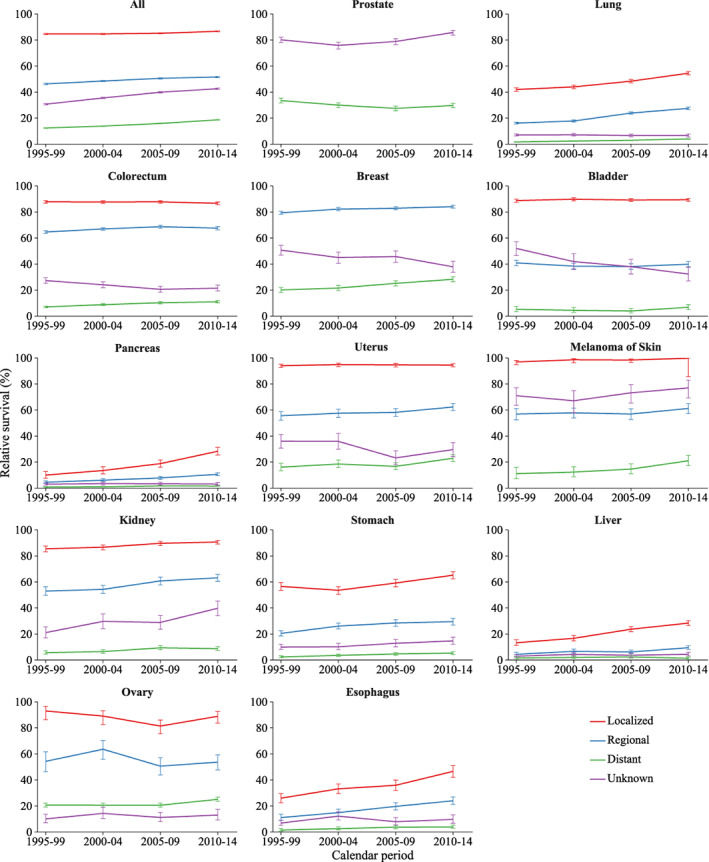
Trends in 5‐year relative survival in elderly patients, by stage and cancer site.

**FIGURE 3:**
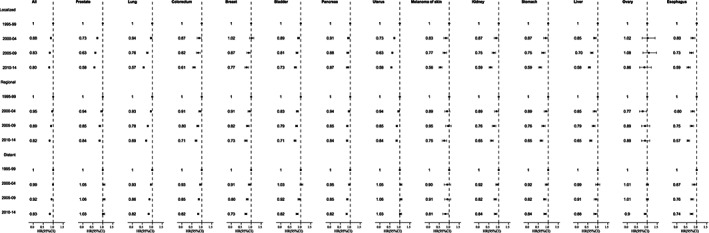
Multivariate‐adjusted hazard ratios for cancer specific death associated with year of diagnosis according to stage, 12 SEER registries, 1995–2014.

Survival gap persists between different stages for elderly cancer patients (Figure 2). Comparing 1995–99 with 2010–14, 5‐year relative survival for all cancers combined increased from 84.7% (84.3–85.1) to 86.7% (86.3–87.0) for localized stage, from 46.3% (45.8–46.8) to 51.6% (51.1–52.1) for regional stage, and from 12.4% (12.1–12.7) to 18.7% (18.4–19.0) for distant stage. The relative survival for early‐stage prostate and breast cancers is close to 100%. Over the study period, the survival for all stages (localized, regional, and distant stage) improved steadily, except for distant prostate and uterus cancer. Furthermore, the degree of improvement in survival was generally greater among the earlier stage groups over the 20‐year period, with about a 42% reduction in liver cancer‐specific deaths among localized patients, compared to only a 12% reduction among distant patients (Figure 3).

**FIGURE 4:**
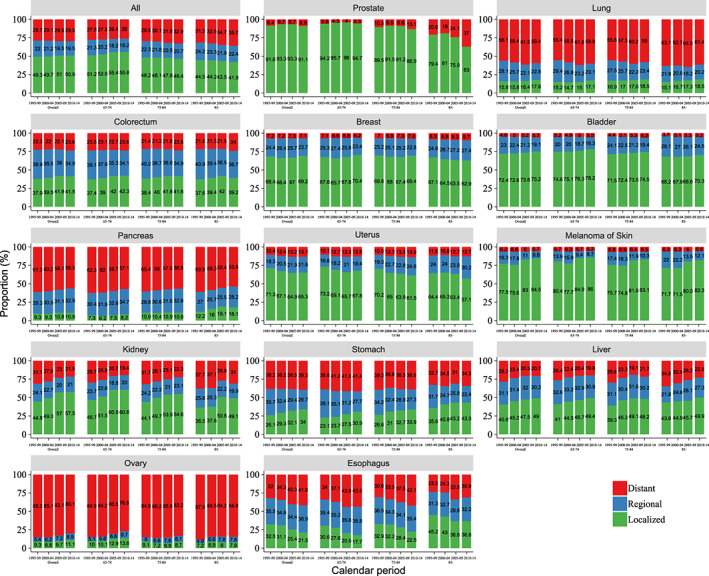
Trends in stage distribution for overall and by age group of elderly cancer patients with known stage at diagnosis.

From 1995 to 2014, the distribution of stages for all cancer combined was relatively stable. The proportion of localized cancer cases increased markedly for kidney, stomach, and liver cancer, but decreased for esophageal cancer (Figure 4; Figure S1). Subgroup analysis showed that the percentage of localized cancer patients was higher in younger patients (65–74 and 75–84 years) than in older patients (85 years or older) for most cancer types, with the exception of lung, pancreatic, stomach, and esophageal cancer. The percentage of localized prostate cancer decreased in patients aged 85 and above. During the study period, the treatment rate for elderly cancer patients was stable, except for pancreatic, stomach, and liver cancer, which had an increasing treatment rate (Figure S2). Subgroup analysis showed the proportion of cancer patients aged 65–74, 75–84, and 85 or older who accepted treatment gradually decreased.

## 4 DISCUSSION

Based on the most recent SEER data from population‐based cancer registries, our study evaluated the survival trend of elderly cancer patients in the United States between 1995 and 2014 and explore the potential causes of such trend. We found that the survival among elderly cancer patients in the United States has continued to increase over the past 20 years, except for patients aged 85 years and above. Given the significant increase in stage‐specific survival, stable treatment rate, and stable distribution of stage, the most likely reason for the improvement in elderly cancer survival is the progress in treatment. These findings could provide a scientific basis for facilitating policy designed to improve cancer survival in the elderly.

Survival for elderly cancer patients improved slightly in the United States from 1995 to 2014. Stage at diagnosis is an important factor that affects the prognosis of cancer patient.^12^, ^13^ Our study found the stable trend for the stage distribution in elderly patients for all cancer combined over two decades, but we did observe an increase in the proportion of localized cancers for cancers of the kidney, stomach, and liver. Screening and improved diagnostic techniques are associated with the detection of early stage of cancers. In the United States, national professional associations advocated for cancer screening for lung, breast, colorectal, cervical, and prostate cancers.^14^ However, routine cancer screenings are only recommended for the younger elderly population (<=75 years old), so the effect of screening on the stage of elderly cancer patients may be limited. This is survival gap consistent with our findings that the proportion of patients in the localized stage of breast, lung, and colorectal cancer has improved slightly. Additionally, the proportion of localized prostate cancer patients over the age of 70 has decreased. This is consistent with the fact that the percentage of men aged 70 and above who had a prostate‐specific antigen (PSA) test decreased from about 52% in 2005 to about 45% in 2014.^15^ The advancement and promotion of diagnostic techniques may partly explain a more favorable stage distribution for kidney, stomach, and liver cancer. For instance, with the availability of diagnostic methods such as endoscopic ultrasound, CT, the positron emission tomography CT, MRI, and laparoscopic staging, the baseline stage of stomach cancer has greatly improved.^16^

Treatment is another key prognostic factor.^14^ Our analyses found that the treatment rate did not change significantly over the periods. However, improvements in elderly cancer survival from 1995 to 2014 were present for almost all stages, indicating that advances in cancer treatment can partially explain these improvements, especially since late‐stage cancer are less likely to be influenced by screening. Survival gains in our study were especially rapid for chronic leukemia and melanoma, most likely due to the development of treatment. It was supported by a previous study, showing that when treated with tyrosine‐kinase inhibitors, most patients with chronic myeloid leukemia experienced a near normal life expectancy.^17^ One previous study found overall mortality rates for melanoma decreased sharply by 17.9% with sharp drops among men aged 50 years above since introduction of new therapies for metastatic melanoma in the United States^18^ The significant improvement in advanced breast cancer survival was consistent with previous studies, demonstrating improved survival with efficient systemic therapies, including the major advances in HER2‐targeted treatment.^19^, ^20^, ^21^ Taken together, our findings indicate that the increase in elderly cancer survival may be primarily due to advances in the treatment, particularly the greater increase in survival for advanced cancers, which was more likely to be attributed to new techniques and new drugs. Further research is warranted to confirm this assertion.

Unsurprisingly, we observed a survival gap by age among elderly cancer patients. This is consistent with our findings that older patients are more likely to be diagnosed at a later stage and receive less treatment, which may account for the survival gap. Meanwhile, we found that the percentage of patients in localized stage increased among those aged 65–74 years, but remained stable even decreased among those aged 75–84 years and aged 85 or older. This might be associated with less screening among older patients.^22^ For breast and colorectal cancer, upper age limit for routine cancer screening was 74 and 75 years, respectively.^23^, ^24^, ^25^ The US Preventive Services Task Force (USPSTF) limits the upper age for heavy current and former smokers to 80 years for lung cancer screening, (the American Cancer Society guideline is 74 years).^14^, ^26^ The USPSTF advises against PSA testing in men aged 70 years and above for prostate cancer.^27^ Furthermore, survival for all cancer improved significantly in the younger old group (65–74 years and 75–84 years) but not in the oldest old (85 years or older) over the study period in the United States, potentially leading to a widening survival gap between the younger and older population. This growing survival gap might relate to less chance to receive curative treatment in the oldest old, and less benefit from advances in novel treatment due to limited life expectancy and more comorbidities for the oldest old than younger old.^28^ However, age alone is not a good index of a person's physiological or functional status, and thus should not be the main factor guiding treatment decisions.^29^, ^30^ Adults aged 85 and above are the fastest‐growing age group in the United States.^22^ The oldest elderly cancer patients, with increased incidence and stable survival, poses a considerable challenge to health care system and deserves greater attention.

Compared with other high‐income countries with comparable health system expenditure, and universal healthcare access,^31^ the survival for elderly cancer patients in the United States were relatively high^10^ (Figures S3 and S4). It suggests that the country has a good level of cancer management, and other countries with worse elderly cancer survival should focus on elderly cancer survival as well. We also found that survival disparities persist among countries for most cancer sites among elderly patients. These countries have disparities may be related to variations in health‐care systems, cancer policy, and clinical treatment among different countries.^32^, ^33^ Fortunately, the elderly cancer survival have consistently improved among these countries over the last two decades.

This study has several limitations. First, the SEER 12 registries only cover about 12.2% of the US population, so its generalizability is unclear. We were also unable to assess various factors that may influence survival, such as frequency of comorbidities, postoperative complications, and emergency presentation. What is more, potential lead‐time bias could be an issue in this study, but we could not eliminate the influence of this bias on the results.

In summary, this study assesses and quantifies the degree of trend in survival for elderly cancer patients in the United States from 1995 to 2014. We observe a slight improvement in the survival of elderly cancer patients in the United States over time, except for those aged 85 years and up. Our results may support that the improvement in elderly cancer survival are more likely related to the progress in cancer treatment in the United States It emphasizes the critical need to promote the age‐friendly healthcare systems and the creation of high‐quality care teams for elderly cancer patients. Future population‐based studies are warrant to explore interventions to improve the elderly cancer survival.

## AUTHOR CONTRIBUTIONS

**Lan An:** Conceptualization (equal); formal analysis (equal); methodology (equal); visualization (equal); writing – original draft (equal). **Ju Wen:** Data curation (equal); supervision (equal); validation (equal); writing – review and editing (equal). **Rongshou Zheng:** Data curation (equal); supervision (equal); validation (equal); writing – review and editing (equal). **Hongmei Zeng:** Data curation (equal); supervision (equal); validation (equal); writing – review and editing (equal). **Siwei Zhang:** Data curation (equal); supervision (equal); validation (equal); writing – review and editing (equal). **Ru Chen:** Data curation (equal); supervision (equal); validation (equal); writing – review and editing (equal). **Kexin Sun:** Data curation (equal); supervision (equal); validation (equal); writing – review and editing (equal). **Li Li:** Data curation (equal); supervision (equal); validation (equal); writing – review and editing (equal). **ShaoMing Wang:** Conceptualization (equal); funding acquisition (equal); supervision (equal); validation (equal); writing – review and editing (equal). **Wenqiang Wei:** Conceptualization (equal); funding acquisition (equal); supervision (equal); validation (equal); writing – review and editing (equal).

## FUNDING INFORMATION

This work was funded by the Chinese Academy of Medical Sciences Initiative for Innovative Medicine [2021‐I2M‐1‐01, 2021‐I2M‐1‐023], the Beijing Nova Program [Z201100006820069].

## CONFLICT OF INTEREST

No potential conflicts of interest are disclosed.

## ETHICS STATEMENT

Ethical approval is not applicable to this study.

## Supporting information


Figure S1.

Figure S2.

Figure S3.

Figure S4.

Table S1.
Table S2

## Data Availability

The data used in this study are available in the National Cancer Institute's Surveillance, Epidemiology, and End Results (SEER) Program at https://seer.cancer.gov/, and the International Cancer Benchmarking Partnership (ICBP) Cancer Survival in High‐Income Countries (SURVMARK‐2) at https://gco.iarc.fr/survival/survmark/.
