# Supplementary figures and images for: Trends in survival for cancer patients aged 65 years or over from 1995 to 2014 in the United States: A population‐based study
Source: Cancer Med. 2022 Nov 10;12(5):6283–93. doi: 10.1002/cam4.5398 (PMC10028112; doi:10.1002/cam4.5398)

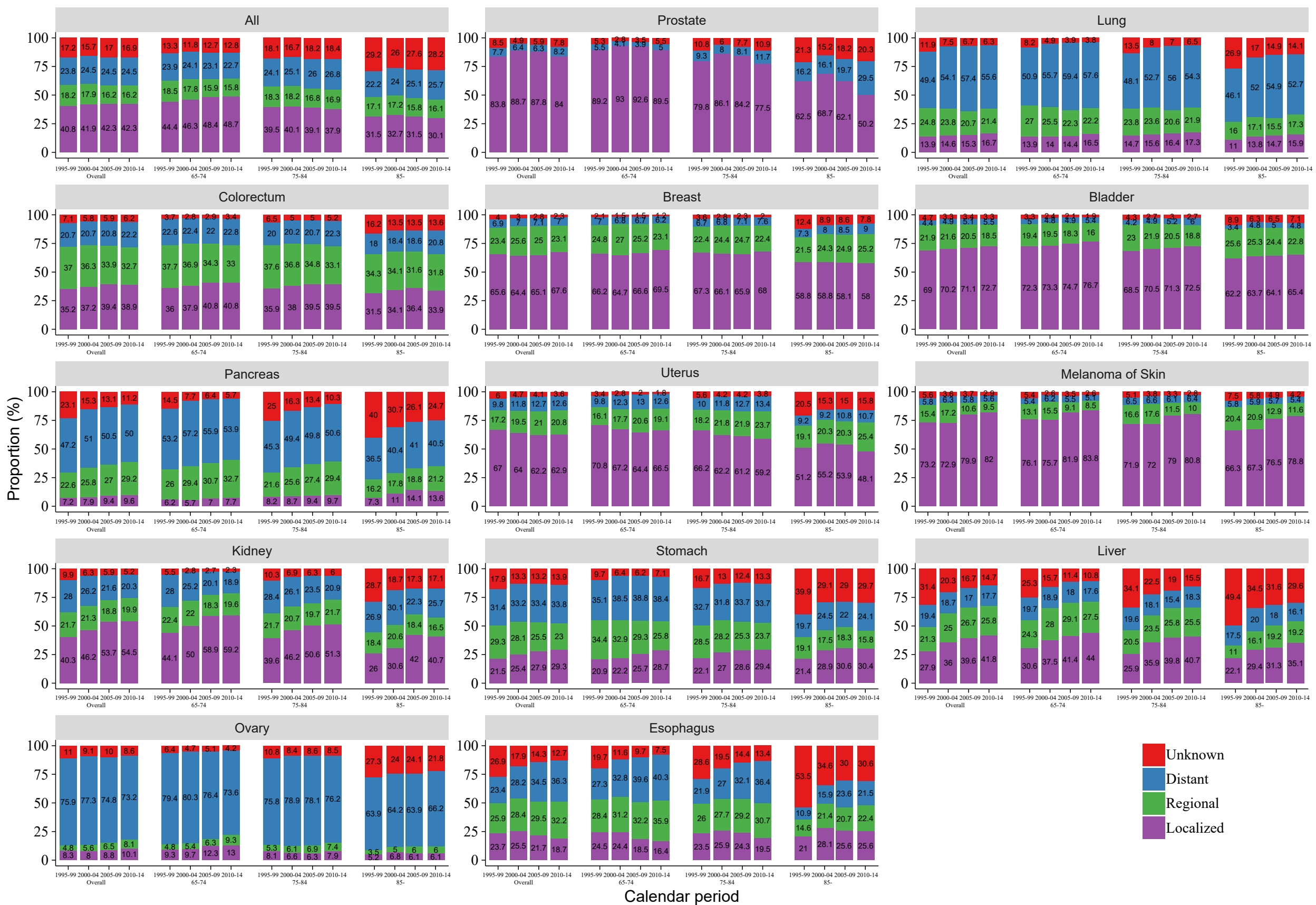

Supplement: Supplementary file 1 — Figure S1. [file CAM4-12-6283-s001.pdf]

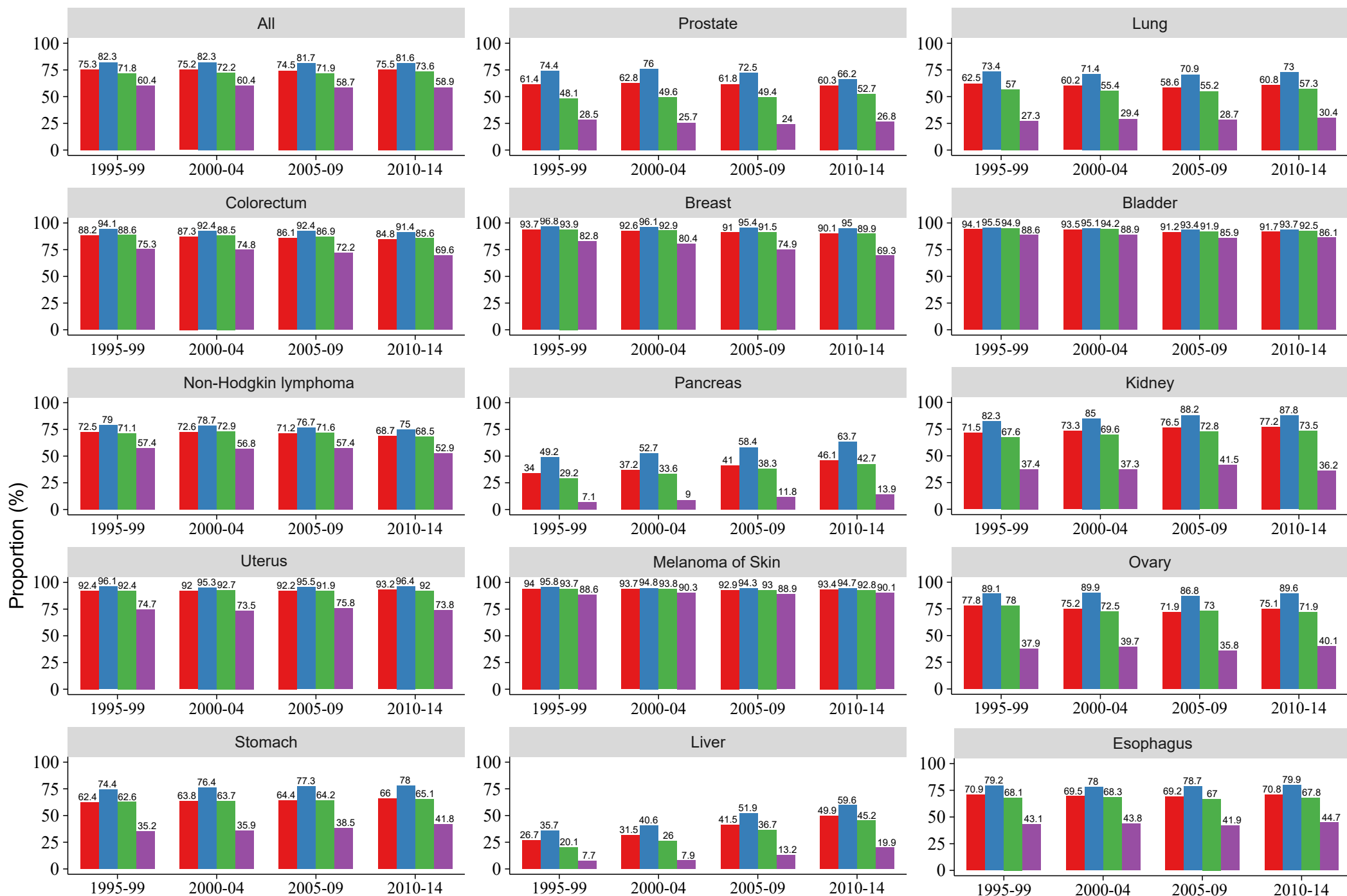

Calendar period

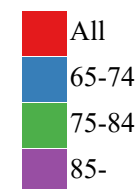

Supplement: Supplementary file 2 — Figure S2. [file CAM4-12-6283-s004.pdf]

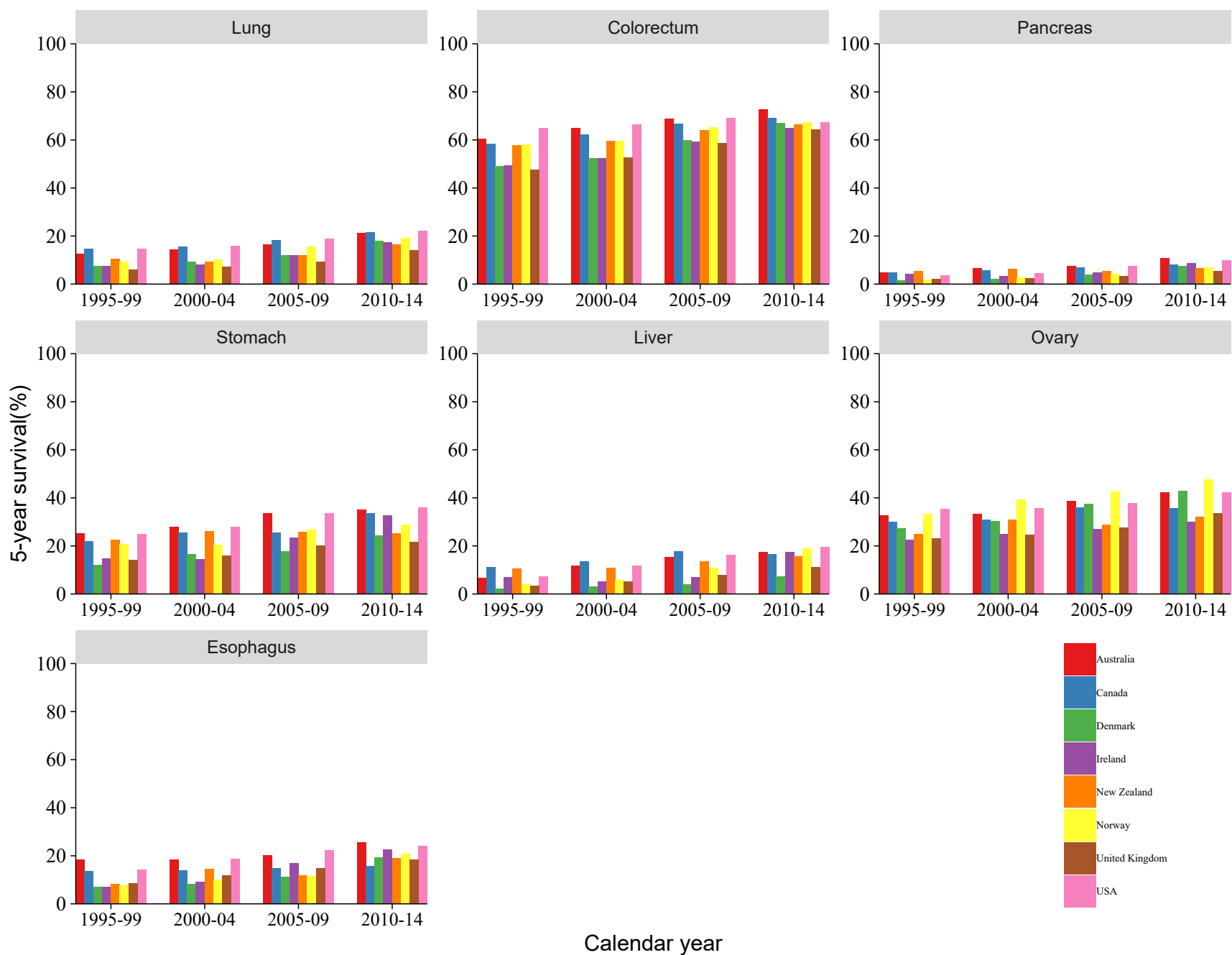

Supplement: Supplementary file 3 — Figure S3. [file CAM4-12-6283-s003.pdf]

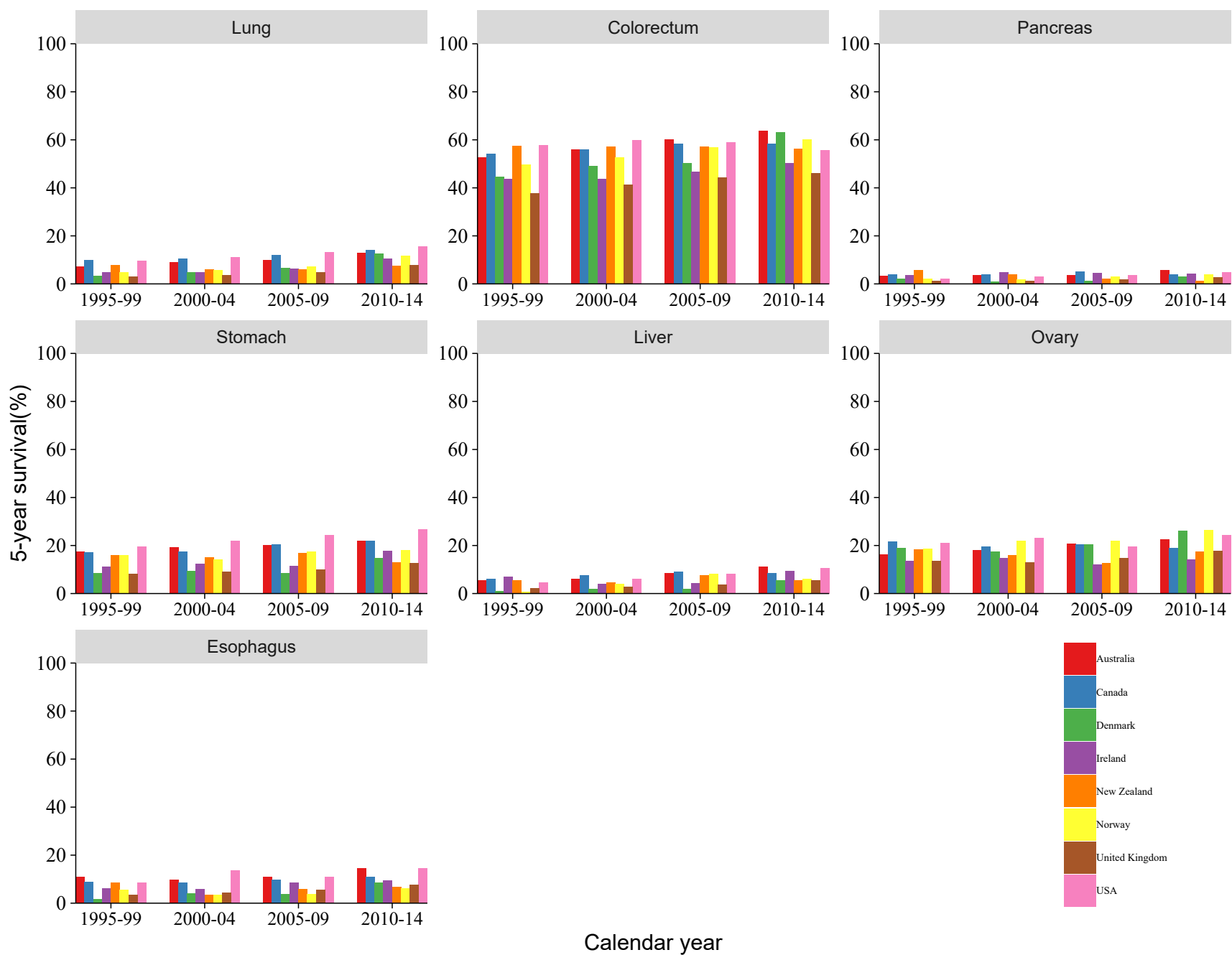

Supplement: Supplementary file 4 — Figure S4. [file CAM4-12-6283-s005.pdf]
