# Supplementary material for: Trends in survival for cancer patients aged 65 years or over from 1995 to 2014 in the United States: A population‐based study
Source: Cancer Med. 2022 Nov 10;12(5):6283–93. doi: 10.1002/cam4.5398 (PMC10028112; doi:10.1002/cam4.5398)
Supplement: Supplementary file 5 — Table S1. Table S2 [file CAM4-12-6283-s002.docx]

**Table S1. Number of patients included in analyses overall, by sex, race, age group, and cancer site**

| Site | All | |  | Age group | | | | | |
| --- | --- | --- | --- | --- | --- | --- | --- | --- | --- |
|  |  |  |  | 65-74 | | 75-84 | | 85- | |
|  | No | P% |  | No | P% | No | P% | No | P% |
| All | 1112441 | 100.0 |  | 537862 | 100.0 | 414641 | 100.0 | 159938 | 100.0 |
| Prostate | 204157 | 18.4 |  | 124719 | 23.2 | 64487 | 15.6 | 14951 | 9.4 |
| Lung | 172876 | 15.5 |  | 82997 | 15.4 | 69159 | 16.7 | 20720 | 13.0 |
| Other ^a^ | 169343 | 15.2 |  | 73595 | 13.7 | 64779 | 15.6 | 30969 | 19.4 |
| Colorectum | 123665 | 11.1 |  | 49974 | 9.3 | 49186 | 11.9 | 24505 | 15.3 |
| Breast | 122401 | 11.0 |  | 61957 | 11.5 | 43737 | 10.6 | 16707 | 10.5 |
| Bladder | 49885 | 4.5 |  | 19680 | 3.7 | 20465 | 4.9 | 9740 | 6.1 |
| Non-Hodgkin lymphoma | 46103 | 4.1 |  | 19822 | 3.7 | 18568 | 4.5 | 7713 | 4.8 |
| Pancreas | 40071 | 3.6 |  | 16668 | 3.1 | 15966 | 3.9 | 7437 | 4.7 |
| Uterus | 27527 | 2.5 |  | 15666 | 2.9 | 9057 | 2.2 | 2804 | 1.8 |
| Melanoma of Skin | 26333 | 2.4 |  | 12705 | 2.4 | 9425 | 2.3 | 4203 | 2.6 |
| Kidney | 25884 | 2.3 |  | 13437 | 2.5 | 9353 | 2.3 | 3094 | 1.9 |
| Stomach | 25702 | 2.3 |  | 10540 | 2.0 | 10346 | 2.5 | 4816 | 3.0 |
| Liver | 20270 | 1.8 |  | 10511 | 2.0 | 7357 | 1.8 | 2402 | 1.5 |
| Chronic Leukemia | 17429 | 1.6 |  | 7169 | 1.3 | 6904 | 1.7 | 3356 | 2.1 |
| Ovary | 16769 | 1.5 |  | 7661 | 1.4 | 6401 | 1.5 | 2707 | 1.7 |
| Esophagus | 12308 | 1.1 |  | 6107 | 1.1 | 4566 | 1.1 | 1635 | 1.0 |
| Acute Leukemia | 11718 | 1.1 |  | 4654 | 0.9 | 4885 | 1.2 | 2179 | 1.4 |

^a^ Other cancers, all remaining cancers except for the sixteen individual cancer listed in the table.

**Table S2. Multivariate-adjusted hazard ratios (HRs) and 95% confidence intervals (CIs) for cancer specific death associated with year of diagnosis according to age group, twelve SEER registries, 1995-2014**

| Site | Age group | 1995-99 | 2000-04 | 2005-09 | 2010-14 | per 5-year increment |
| --- | --- | --- | --- | --- | --- | --- |
| All | 65-74 | 1.00 | 0.92(0.91-0.93) | 0.80(0.79-0.81) | 0.70(0.69-0.71) | 0.89(0.88-0.89) |
|  | 75-84 | 1.00 | 0.97(0.96-0.98) | 0.93(0.92-0.95) | 0.88(0.86-0.89) | 0.96(0.95-0.96) |
|  | 85+ | 1.00 | 1.04(1.01-1.06) | 1.04(1.02-1.07) | 1.04(1.02-1.07) | 1.01(1.00-1.02) |
|  | 65-74 | 1.00 | 0.92(0.85-1.01) | 0.78(0.72-0.86) | 0.66(0.61-0.73) | 0.87(0.85-0.90) |
| Acute Leukemia | 75-84 | 1.00 | 0.97(0.90-1.06) | 0.90(0.82-0.98) | 0.79(0.73-0.87) | 0.93(0.90-0.95) |
|  | 85+ | 1.00 | 0.95(0.83-1.08) | 0.84(0.74-0.96) | 0.83(0.73-0.94) | 0.93(0.90-0.97) |
| Bladder | 65-74 | 1.00 | 0.92(0.85-1.00) | 0.87(0.80-0.95) | 0.72(0.66-0.78) | 0.90(0.88-0.93) |
|  | 75-84 | 1.00 | 0.98(0.91-1.06) | 1.05(0.98-1.13) | 0.91(0.85-0.99) | 0.98(0.96-1.01) |
|  | 85+ | 1.00 | 0.94(0.85-1.04) | 0.97(0.88-1.07) | 0.97(0.88-1.07) | 1.00(0.97-1.03) |
| Breast | 65-74 | 1.00 | 0.80(0.76-0.85) | 0.69(0.65-0.73) | 0.57(0.54-0.60) | 0.83(0.82-0.85) |
|  | 75-84 | 1.00 | 0.94(0.89-1.00) | 0.91(0.85-0.96) | 0.82(0.77-0.88) | 0.94(0.92-0.96) |
|  | 85+ | 1.00 | 1.12(1.02-1.23) | 1.08(0.99-1.19) | 1.15(1.05-1.26) | 1.04(1.01-1.07) |
|  | 65-74 | 1.00 | 0.71(0.64-0.79) | 0.48(0.42-0.53) | 0.31(0.27-0.35) | 0.68(0.65-0.70) |
| Chronic Leukemia | 75-84 | 1.00 | 0.76(0.68-0.84) | 0.61(0.55-0.68) | 0.48(0.42-0.54) | 0.78(0.76-0.81) |
|  | 85+ | 1.00 | 0.92(0.79-1.07) | 0.75(0.64-0.87) | 0.60(0.52-0.71) | 0.84(0.80-0.88) |
| Colorectum | 65-74 | 1.00 | 0.90(0.87-0.94) | 0.75(0.72-0.78) | 0.70(0.67-0.73) | 0.88(0.87-0.89) |
|  | 75-84 | 1.00 | 0.95(0.92-0.99) | 0.90(0.87-0.94) | 0.87(0.84-0.91) | 0.95(0.94-0.97) |
|  | 85+ | 1.00 | 1.02(0.96-1.07) | 1.06(1.00-1.12) | 1.10(1.03-1.16) | 1.03(1.01-1.05) |
| Uterus | 65-74 | 1.00 | 1.02(0.92-1.12) | 0.97(0.89-1.07) | 0.80(0.72-0.87) | 0.92(0.90-0.95) |
|  | 75-84 | 1.00 | 0.86(0.77-0.96) | 0.90(0.81-1.01) | 0.83(0.75-0.93) | 0.95(0.92-0.98) |
|  | 85+ | 1.00 | 1.15(0.95-1.40) | 0.93(0.77-1.13) | 0.95(0.78-1.15) | 0.96(0.91-1.02) |
| Esophagus | 65-74 | 1.00 | 0.84(0.77-0.92) | 0.68(0.62-0.75) | 0.60(0.55-0.66) | 0.84(0.82-0.86) |
|  | 75-84 | 1.00 | 0.84(0.76-0.94) | 0.82(0.73-0.91) | 0.67(0.60-0.75) | 0.88(0.86-0.92) |
|  | 85+ | 1.00 | 0.78(0.63-0.97) | 0.85(0.69-1.05) | 0.78(0.63-0.96) | 0.95(0.89-1.01) |
| Kidney | 65-74 | 1.00 | 0.91(0.84-0.98) | 0.76(0.70-0.82) | 0.71(0.65-0.77) | 0.89(0.86-0.91) |
|  | 75-84 | 1.00 | 0.91(0.83-0.99) | 0.76(0.70-0.84) | 0.68(0.62-0.75) | 0.88(0.85-0.90) |
|  | 85+ | 1.00 | 0.89(0.76-1.06) | 0.95(0.81-1.11) | 0.93(0.79-1.09) | 0.99(0.94-1.04) |
| Liver | 65-74 | 1.00 | 0.88(0.82-0.95) | 0.77(0.72-0.83) | 0.64(0.59-0.68) | 0.86(0.84-0.88) |
|  | 75-84 | 1.00 | 0.92(0.84-1.01) | 0.80(0.73-0.88) | 0.74(0.67-0.80) | 0.90(0.88-0.92) |
|  | 85+ | 1.00 | 0.97(0.80-1.16) | 0.90(0.76-1.08) | 0.78(0.66-0.92) | 0.92(0.87-0.96) |
| Lung | 65-74 | 1.00 | 0.92(0.90-0.94) | 0.82(0.80-0.83) | 0.73(0.72-0.75) | 0.90(0.89-0.91) |
|  | 75-84 | 1.00 | 0.93(0.91-0.95) | 0.83(0.81-0.85) | 0.77(0.75-0.79) | 0.91(0.91-0.92) |
|  | 85+ | 1.00 | 0.97(0.92-1.03) | 0.87(0.83-0.92) | 0.85(0.80-0.89) | 0.94(0.93-0.95) |
| Non-Hodgkin lymphoma | 65-74 | 1.00 | 0.91(0.84-0.98) | 0.76(0.70-0.82) | 0.71(0.65-0.77) | 0.89(0.86-0.91) |
|  | 75-84 | 1.00 | 0.91(0.83-0.99) | 0.76(0.70-0.84) | 0.68(0.62-0.75) | 0.88(0.85-0.90) |
|  | 85+ | 1.00 | 0.89(0.76-1.06) | 0.95(0.81-1.11) | 0.93(0.79-1.09) | 0.99(0.94-1.04) |
| Ovary | 65-74 | 1.00 | 1.04(0.97-1.12) | 0.98(0.91-1.06) | 0.85(0.79-0.92) | 0.95(0.93-0.97) |
|  | 75-84 | 1.00 | 0.95(0.88-1.03) | 1.02(0.94-1.11) | 0.91(0.83-0.99) | 0.98(0.96-1.01) |
|  | 85+ | 1.00 | 1.05(0.91-1.21) | 0.97(0.85-1.12) | 0.92(0.80-1.06) | 0.97(0.93-1.01) |
| Pancreas | 65-74 | 1.00 | 0.91(0.87-0.96) | 0.79(0.75-0.83) | 0.68(0.65-0.72) | 0.88(0.86-0.89) |
|  | 75-84 | 1.00 | 0.91(0.86-0.96) | 0.84(0.80-0.89) | 0.79(0.75-0.83) | 0.92(0.91-0.94) |
|  | 85+ | 1.00 | 0.98(0.89-1.08) | 0.95(0.87-1.04) | 0.94(0.86-1.02) | 0.98(0.95-1.00) |
| Prostate | 65-74 | 1.00 | 0.77(0.74-0.80) | 0.67(0.64-0.70) | 0.62(0.59-0.66) | 0.84(0.83-0.86) |
|  | 75-84 | 1.00 | 0.88(0.84-0.92) | 0.80(0.76-0.84) | 0.83(0.79-0.88) | 0.93(0.91-0.94) |
|  | 85+ | 1.00 | 0.90(0.83-0.99) | 0.96(0.88-1.05) | 1.03(0.94-1.13) | 1.02(0.99-1.05) |
| Melanoma of Skin | 65-74 | 1.00 | 0.81(0.72-0.92) | 0.71(0.63-0.81) | 0.53(0.47-0.60) | 0.82(0.79-0.85) |
|  | 75-84 | 1.00 | 0.84(0.73-0.95) | 0.93(0.82-1.06) | 0.71(0.62-0.82) | 0.91(0.88-0.95) |
|  | 85+ | 1.00 | 1.13(0.90-1.41) | 1.09(0.87-1.35) | 0.94(0.76-1.16) | 0.96(0.91-1.03) |
| Stomach | 65-74 | 1.00 | 0.91(0.85-0.97) | 0.74(0.69-0.79) | 0.67(0.63-0.72) | 0.87(0.85-0.89) |
|  | 75-84 | 1.00 | 0.89(0.83-0.96) | 0.83(0.77-0.89) | 0.72(0.67-0.77) | 0.90(0.88-0.92) |
|  | 85+ | 1.00 | 1.11(0.98-1.25) | 1.00(0.88-1.12) | 0.97(0.86-1.09) | 0.98(0.94-1.01) |
